# Ultrastructural Variations of Antennae and Labia Are Associated with Feeding Habit Shifts in Stink Bugs (Heteroptera: Pentatomidae)

**DOI:** 10.3390/biology10111161

**Published:** 2021-11-10

**Authors:** Xinyu Li, Li Tian, Hu Li, Wanzhi Cai

**Affiliations:** Department of Entomology, MOA Key Lab of Pest Monitoring and Green Management, College of Plant Protection, China Agricultural University, Beijing 100193, China; lixinyu91@cau.edu.cn (X.L.); ltian@cau.edu.cn (L.T.); lih@cau.edu.cn (H.L.)

**Keywords:** Pentatomidae, mouthpart, antenna, morphology, ultrastructure, adaptation

## Abstract

**Simple Summary:**

Stink bugs (Pentatomidae) are one of the most diverse insect groups in suborder Heteroptera (Hemiptera). They are either plant feeders or predators, comprising series of invasive agricultural pests and natural enemies with great economic importance. Species of stink bugs possess various antennal and mouthpart traits apparently related with feeding habits, but key morphological traits associated with feeding habit shift remain largely unexploited. In this study, we compare the ultrastructures of antennae, labia, and mandibular and maxillary stylets of 17 pentatomid species, representing both phytophagous and predatory species chosen from four subfamilies. We identify a set of key ultrastructural characteristics closely associated with feeding habit transition in stink bugs and discuss their adaptative implications in an evolutionary aspect.

**Abstract:**

The family Pentatomidae (stink bugs) is one of the largest groups in Heteroptera, containing many important pests and natural enemies. They exhibit highly diversified feeding habits and related structural modifications, but the key morphological characteristics associated with feeding habit radiation remain unclear. In the current study, we address this question by analyzing morphological variations of feeding related organs. We compare the ultrastructures of antennae and mouthparts across the chosen 17 species in Pentatomidae, representing both plant feeders and predators from four subfamilies. A strong association between ultrastructural adaptation and feeding habit transition has been revealed. The long, sharp, and hook-like mandibular teeth and maxillary barbs are exclusively present in predatory Pentatomidae, suggesting their tight association with the shift of feeding habit from phytophagy to predation. Significant differences between phytophagous and predatory species are also found in antennal and labial sensilla types and arrangements, implying their important function in food selection. Our data identify a series of key morphological structures associated with feeding habit variations among stink bugs, which will facilitate future studies on adaptive evolution of feeding habits, utilization, and population control of economic species in Pentatomidae as well as in other heteropteran lineages.

## 1. Introduction

Heteroptera (commonly known as true bugs) are the largest group with great morphological and biological diversifications in Hemiptera. They are distributed worldwide, inhabiting various natural and agricultural habitats (e.g., terrestrial and aquatic; shrublands, forests, and farmlands) and exhibiting significantly diversified feeding habits [1,2,3]. Adaptations in feeding-associated morphology have been shown among true bugs with distinct food preferences [4,5,6,7]. For instance, predatory heteropterans tend to have stout and strong labium, enclosing highly serrated maxillae fringed with sharp teeth [4,5,8], whereas herbivorous lineages often have slim and slender labia, possessing smooth-edged maxillae [4,9,10]. Substantial modifications of antennae have been documented to be related to variations in feeding habits, displaying a large number of mechanoreceptors in plant feeders [11], and specific types of antennal sensilla existed predominantly in predators [12]. Consequently, it is hypothesized that the evolutionary radiation of true bugs has been partially driven by a shift in feeding habit and a modification of feeding-associated morphology [2,4,13,14]. However, a lack of comparative analysis of the key morphological adaptations in the context of phylogeny is an obstacle to further our understanding of heteropteran evolution [15].

Pentatomidae Leach is one of a few heteropteran families containing both plant feeders and predators and hence is a good example for characterizing key morphological changes along with feeding habit transition [16]. Despite most Pentatomidae being phytophagous insects feeding on a wide range of plants, the subfamily Asopinae Amyot & Serville are generalist predators, preying on a large variety of arthropods [3,17]. Diversified feeding habits not only contribute to a broad ecological adaptation of stink bugs but also tied them closely with agriculture and forestry. Some plant feeding species are important invasive and agricultural pests worldwide, e.g., *Halyomorpha halys* (Stål, 1855) [18], damaging numerous crops and fruits due to direct feeding or transmission of plant pathogens [19,20,21,22,23], while some predatory species, e.g., *Eocanthecona furcellata* (Wolff, 1811) [24], have become promising biological control agents against lepidopteran and coleopteran pests [17,25]. As a result, many pentatomid species have received considerable attention due to their great economic importance ([26] and pp. 681–756 in [3]). Nonetheless, we have limited knowledge about key morphological traits associated with feeding habit variation in this group. By far, ultrastructures of feeding related organs in pentatomids have only been described in a few polyphagous species in Pentatominae Leach and Asopinae [27,28,29,30]. Other subfamilies, especially those representing oligophagous feeding pattern, are still largely unexplored. Such limited and biased taxa sampling impedes our understanding on how morphological evolution is associated with feeding habits transition in this insect group.

In the present study, we compared the ultrastructures of the mouthparts and antennae of 17 species from 4 subfamilies in Pentatomidae, representing phytophagous (oligophagy and polyphagy) and predatory (polyphagy) feeding habits, and discussed key morphological modifications associated with the variation of feeding habits in the aspect of phylogeny.

## 2. Materials and Methods

### 2.1. Taxon Sampling

A total of 17 pentatomid species, representing plant feeders and predators from 4 subfamilies, Asopinae, Pentatominae, Phyllocephalinae and Podopinae, were included in a comparative morphological and phylogenetic analysis (Table S1). The sample comprises 6 species characterized in the present study, namely *Cressona divaricata* Zheng & Zou, 1982 [31]—Phyllocephalinae Amyot & Serville; *Eo. furcellata*—Asopinae; *Eurydema dominulus* (Scopoli, 1763) [32]; *H. halys*, *Plautia crossota* (Dallas, 1851) [33]—Pentatominae; and *Scotinophara lurida* (Burmeister, 1834) [34]—Podopinae Amyot & Serville, and another 11 species with their relevant ultrastructures documented previously (Table S1). Adult specimens examined in this study were collected from several localities in China in 2012–2020 (Table S2) and preserved in 95% ethanol. Three males and three females of each species were prepared for morphological examination except that a male and a female of *C. divaricata* were examined due to the small amount of available specimens. Host plant information of the sampled pentatomid species was summarized from publications and is provided in Table S3.

### 2.2. Sample Preparation for Scanning Electronic Microscopy

Antennae, labia, and stylet bundles of the examined specimens were removed from the heads, immersed in PBS buffer (pH = 7.4), and cleaned in an ultrasonic cleaner (30 °C, 30 s). The samples were then dehydrated through an ascending series of ethanol (70%, 80%, 85%, 90%, 95%, and 100% ethanol, 15 min in each), followed by drying in CO_2_ using a Leica EM CPD300 critical point dryer. The prepared samples were then mounted on aluminum stubs using double-side carbon adhesive tape, coated with platinum, and examined using a HITACHI SU8010 scanning electronic microscope at the Institute of Microbiology, Chinese Academy of Sciences (Beijing, China). The length, basal diameter, and distribution of antennal and labial sensilla are provided in Tables S4–S9, and the average density of sensilla basiconica on antennal distiflagellomere are provided in Table S10. The morphological terminology follows Schuh and Weirauch [2], Cobben [4], and Schneider [35].

### 2.3. DNA Extraction, Molecular Marker Amplification, and Sequencing

Fragments of two mitochondrial genes, *cox1* and *rrnL*, were used as genetic markers for phylogenetic analysis. Sequences of these two genes of *C. divaricata* were sequenced in the present study, and that of 16 species (except for *C. divaricata*) were retrieved from GenBank (Table S1). The total genomic DNA of *C. divaricata* was extracted from thoracic muscle tissues from an adult specimen using the TIANamp Genomic DNA Kit (TIANGEN, Beijing, China). The remaining body parts were retained as vouchers and deposited in China Agricultural University. Mitochondrial fragments were amplified using the universal insect mitochondrial primers C1-J1709 (5′-AATTGGWGGWTTYGGAAAYTG-3′), C1-N2776 (5′-GGTAATCAGAGTATCGWCGNGG-3′), LR-J12888 (5′-CCGGTCTGAACTCAGATCATGTA-3′), and LR-N13889 (5′-ATTTATTGTACCTTTTGTATCAG-3′) [36]. The PCR reactions and amplification conditions were conducted following Li et al. [37]. Purification and sequencing of PCR products were performed by Tsingke Biotechnology Co., Ltd., Beijing, China. The sequences were assembled using SeqMan Pro v. 7.1.0 (DNASTAR Inc., Maddison, WI, USA), aligned by MAFFT v. 7 online service [38,39] with default parameters, and deposited in GenBank with the accession numbers MZ673416 and MZ676042 (Table S1).

### 2.4. Phylogenetic Analysis

The phylogenetic relationships of all 17 Pentatomidae species were analyzed based on the concatenated matrix of *cox1* and *rrnL* using maximum likelihood (ML) on the IQ-TREE web server [40]. The matrix was partitioned by genes, and the best-fit partitioning schemes and substitution models were selected using the “Auto” option, resulting in TIM2+F+I+G4 for *cox1* and GTR+F+I+G4 for *rrnL*. A 1000 ultrafast bootstrap approximation [41] was performed to evaluate the support values for branches. A phylogenetic hypothesis about the chosen species in each subfamily forming a monophyletic group was mainly adopted from Rider et al. [42] and treated as a constraint for tee search. Species from Largidae Amyot & Serville, Rhopalidae Amyot & Serville, and Reduviidae Latreille were selected as outgroups [13,14,43]. Ancestral character state reconstruction (ASR) was performed using the maximum parsimony method in Mesquite 3.51 [44]. Information on the morphological characteristics used for ASR was obtained from the present study and previous publications, summarized and coded in Table S11.

## 3. Results and Discussion

### 3.1. Conserved Morphology and Sensilla Type of Antenna in Stink Bugs

**Figure 1:**
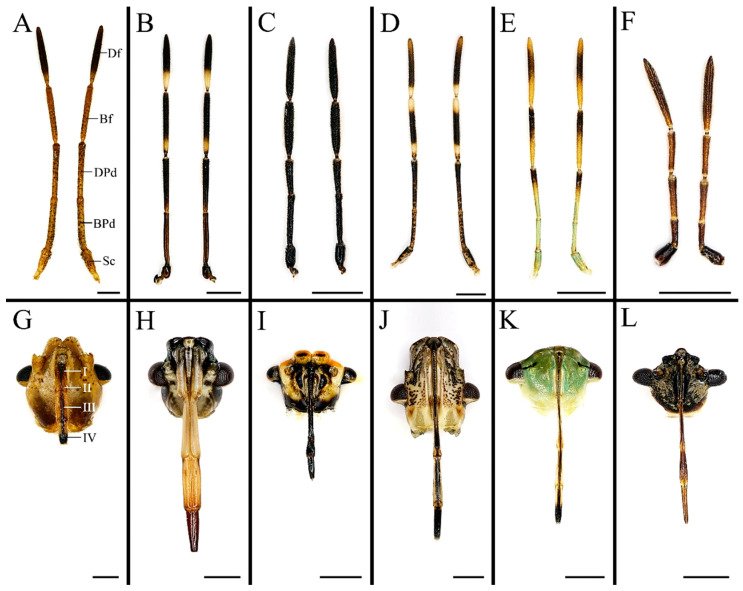
Antennae and labia of six pentatomid species examined in present study. (**A**,**G**) *Cressona divaricata*, (**B**,**H**) *Eocanthecona furcellata*, (**C**,**I**) *Eurydema dominulus*, (**D**,**J**) *Halyomorpha halys*, (**E**,**K**) *Plautia crossota*, and (**F**,**L**) *Scotinophara lurida*. Scale bars: (**A**–**L**) 1 mm. Abbreviations: Bf, basiflagellomere; BPd, basal pedicel; Df, distiflagellomere; DPd, distal pedicel; and Sc, scape.

**Figure 2:**
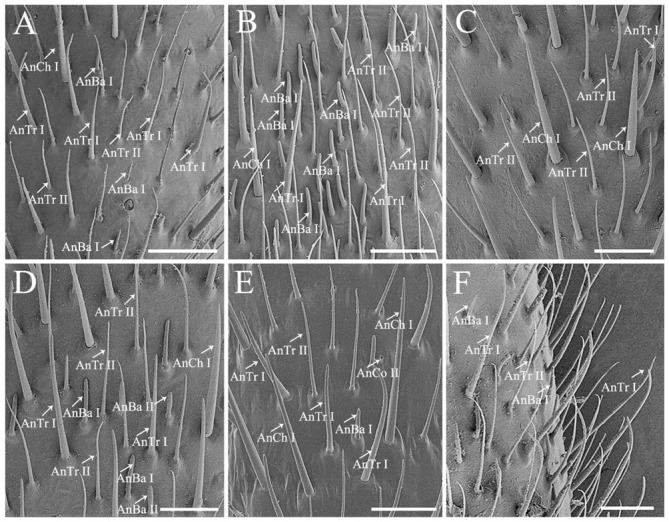
Dorsal view of the antennal distiflagellomere of the six pentatomid species, showing the distribution of antennal sensilla chaetica, sensilla trichodea, and sensilla basiconica. (**A**) *Cressona divaricata*, (**B**) *Eocanthecona furcellata*, (**C**) *Eurydema dominulus*, (**D**) *Halyomorpha halys*, (**E**) *Plautia crossota*, and (**F**) *Scotinophara lurida*. Scale bars: (**A**) 50 μm; (**B**–**F**) 25 μm. Abbreviations: AnBa I–II, antennal sensilla basiconica I–II; AnCh I, antennal sensilla chaetica I; AnTr I–II, antennal sensilla trichodea I–II; and AnCo II, antennal sensilla coeloconica II.

**Figure 3:**
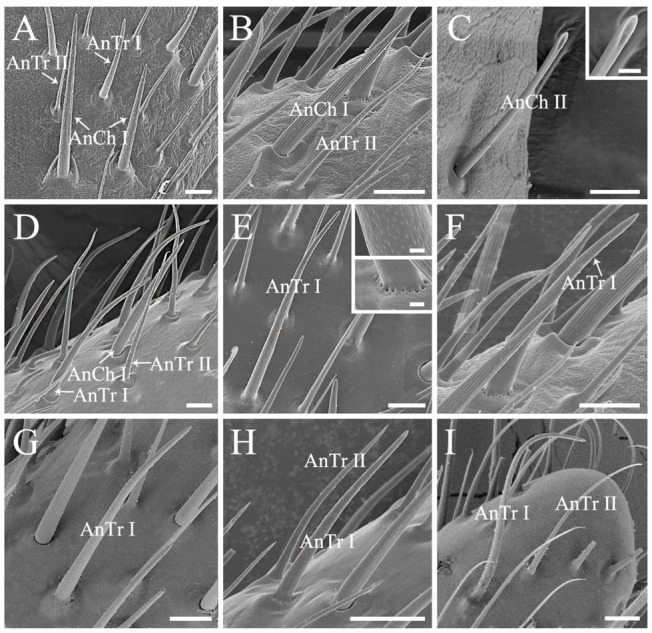
Antennal sensilla chaetica and sensilla trichodea of the six pentatomid species. (**A**,**B**,**F**) *Eurydema dominulus*; (**C**,**E**) *Eocanthecona furcellata*, showing the spoon shaped apex of Ch II in (**C**); (**D**) *Cressona divaricata*; (**G**) *Halyomorpha halys*; (**H**) *Plautia crossota*; and (**I**) *Scotinophara lurida*. Scale bars: (**A**–**G**,**I**) 10 μm, (**C**) 2.5 μm, and (**E**) 0.5 μm; (**H**) 15 μm. Abbreviations: AnCh I–II, antennal sensilla chaetica I–II; AnTr I–II, antennal sensilla trichodea I–II.

**Figure 4:**
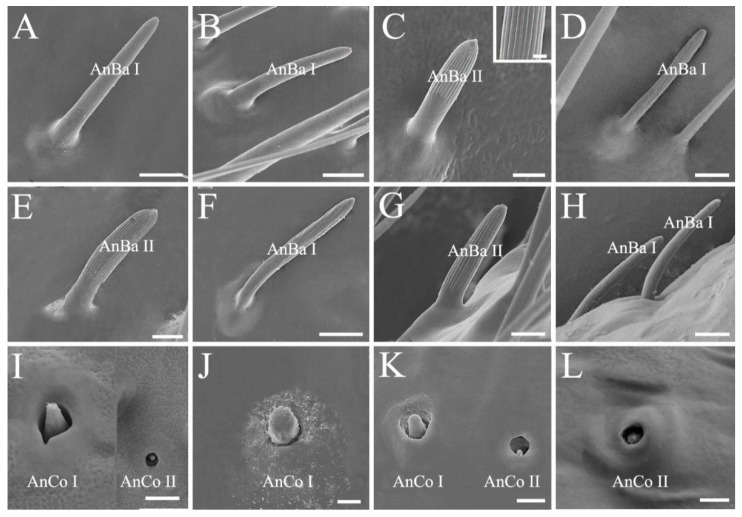
Antennal sensilla basiconica and sensilla coeloconica of the six pentatomid species. (**A**) *Cressona divaricata*; (**B**,**I**) *Eocanthecona furcellata*; (**C**) *Eurydema dominulus*, with grooves shown in the ox; (**D**,**E**,**J**) *Halyomorpha halys*; (**F**,**G**,**K**) *Plautia crossota*; and (**H**,**L**) *Scotinophara lurida*. Scale bars: (**A**,**B**,**D**,**F**,**H**) 5 μm, (**C**,**E**,**G**,**I**–**L**) 2.5 μm, and (**C**) 0.5 μm. Abbreviations: AnBa I–II, antennal sensilla basiconica I–II; AnCo I–II, antennal sensilla coeloconica I–II.

Insect antennae carry distinct types of olfactory sensilla that play various biological functions, such as orientation, foraging, and inter/intraspecific communication [28,39,40]. We found that antennal morphology and sensillum type are similar among the species of stink bugs we examined (Figure 1 and Figure 2). The antennae of all 17 Pentatomidae are a pair of long and flexible sensory appendages, ventrolaterally located on the head, consisting of five segments: a proximal scape, a subdivided pedicel forming a basal segment and a distal segment, a basiflagellomere, and a distiflagellomere [11,12]. All of the segments are approximately cylindrical except the apex of distiflagellomere abruptly narrowed. We found four types of sensilla, namely sensilla chaetica, sensilla trichodea, sensilla basiconica, and sensilla coeloconica (Figure 2, Figure 3 and Figure 4) [27,29]. They showed a conspicuous density gradient with the sensillum number increasing from antennal scape to distiflagellomere. In the six species examined in present study, *C. divaricata* and *S. lurida* have their antennal sensilla primarily located on the distiflagellomere, while the rest of species have most of their sensilla located on basi- and distiflagellomeres. The length, basal diameter, and distribution of these sensilla of the six species are provided in Tables S4–S9. The average density of basiconica sensilla on antennal distiflagellomere is summarized in Table S10.

Antennal sensilla chaetica (AnCh) are stiff and strong, with deep grooves and well-developed sockets, representing one of the two dominant antennal sensillum types in stink bugs (Figure 2 and Figure 3) [11,29]. Two subtypes of Ch can be distinguished among all of the sampled species. Antennal sensilla chaetica I (AnCh I) are sharp tipped, located on all of the five antennal segments in the six examined pentatomid species (Figure 2 and Figure 3A,B,D). Antennal sensilla chaetica II (AnCh II) possess spoon shaped tips and are only detected on antennal scape and basal pedicel in *Eo*. *furcellata* (Figure 3C).

Antennal sensilla trichodea (AnTr) are slender and hair-like structures with tapered tips, acting as the other dominant type of antennal sensilla in pentatomids (Figure 2 and Figure 3) [11,29]. Two subtypes of Tr can be distinguished. Antennal sensilla trichodea I (AnTr I) are longer and broader, possessing more distinct minute wall-pores (Figure 3E) than antennal sensilla trichodea II (AnTr II). In *Eo. furcellata* and *Eu. dominulus*, the bases of AnTr I are circled by evident pores (Figure 3E,F), while the bases of AnTr I are smooth and poreless in the other four species (Figure 3D,G–I). Antennal sensilla trichodea I and II are variously distributed in different species. In the six examined species, they are widely spread from the basal part of the pedicel to the distal end of distiflagellomere in *H. halys*. In *Eo. furcellata* and *P. crossota*, these organs can be found mainly on the distal subdivision of the pedicel to distiflagellomere. In *C. divaricata*, *Eu. dominulus*, and *S. lurida*, AnTr I and II are mostly distributed on the basiflagellomere and distiflagellomere.

Antennal sensilla basiconica (AnBa) are peg-shaped. Two subtypes of Ba can be distinguished. Antennal sensilla basiconica I (AnBa I) and II (AnBa II) have abruptly blunt tips and straight longitudinal grooves exceeding half length of the sensilla (Figure 2 and Figure 4A–H). Antennal sensilla basiconica I are long and narrow, and AnBa II are short and broad. Both of them are located from the distal subdivision of antennal pedicel to distiflagellomere in *Eo*. *furcellata*, *H*. *halys*, and *P*. *crossota* but are mainly distributed on the two flagellomeres in *C. divaricata*, *Eu. dominulus*, and *S. lurida*. Sensilla basiconica are shorter in length and fewer in number than sensilla trichodea. Notably, a much higher density of sensilla basiconica is observed on distiflagellomere in predatory *Eo*. *furcellata* than in the phytophagous stink bugs (Figure 2; Table 1 and Table S10). Antennal sensillum basiconica is the predominant sensillum type responsible for food odor reception for insects [45,46], and hence, the increased number of AnBa in predators might be associated with prey orientation and selection.

Antennal sensilla coeloconica (AnCo) are cone-shaped and situated in cavities (Figure 4I–L). They are smaller and sparser than other types of sensilla. This type of sensilla is only found on the antennae of *Eo*. *furcellata*, *H*. *halys*, *P*. *crossota*, and *S*. *lurida* but absent on the other examined pentatomid species. Two subtypes of Co can be distinguished. Antennal sensilla coeloconica I (AnCo I) are larger in size, with the tips slightly elevated above the antennal surface and found in *Eo*. *furcellata*, *H*. *halys*, and *P*. *crossota* (Figure 4I–K); AnCo II are sunken deeply, with the tips below the surface and observed in *Eo*. *furcellata*, *P*. *crossota*, and *S*. *lurida* (Figure 4I,K,L).

Antenna and its sensilla of insects are various in shape and structure, which are suggested to be shaped by adaptive evolution to detect certain odor molecules [35,45,47]. In the present study, we found that the general antennal morphology and sensillum types are conserved in stink bugs (Table 1), which reflects constraints imposed by phylogenetic relationships on basic architecture of antenna. Further study involved with molecular mechanisms of olfactory detection in Pentatomidae is in demand to understand antennal sensilla construction and function across stink bugs with varied feeding habits.

### 3.2. Ultrastructural Variations of the Labia between Species with Different Feeding Habits

**Figure 5:**
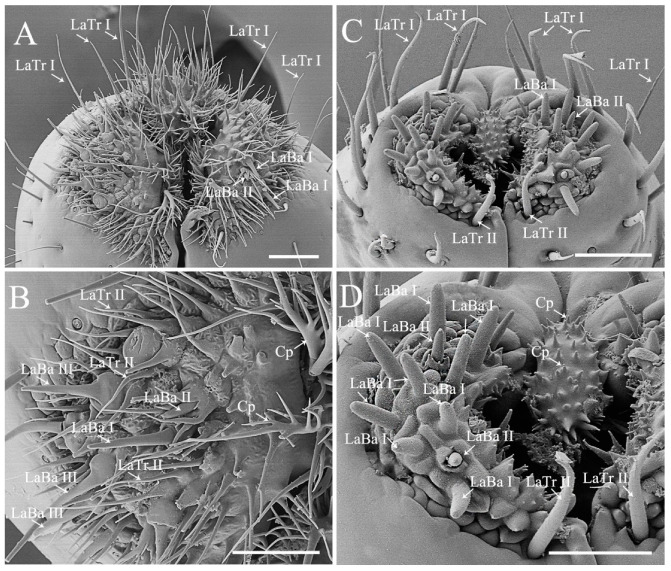
Labial apex of *Eocanthecona furcellata* (**A**,**B**) and *Eurydema dominulus* (**C**,**D**), showing labial sensilla and cuticular projections. Scale bars: (**A**,**C**) 30 μm; (**B**,**D**) 20 μm. Abbreviations: Cp, cuticular projection; LaBa III–V, labial sensilla basiconica III–V; and LaTr I–II, labial sensilla trichodea I–II.

**Figure 6:**
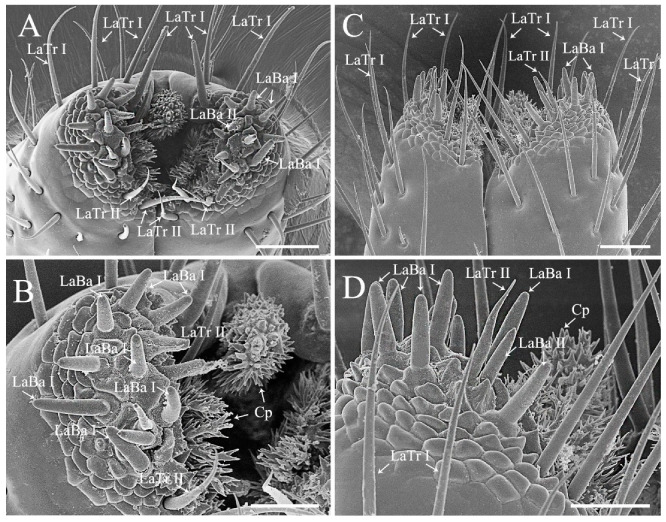
Labial apex of *Halyomorpha halys* (**A**,**B**) and *Plautia crossota* (**C**,**D**), showing labial sensilla and cuticular projections. Scale bars: (**A**,**C**) 25 μm; (**B**,**D**) 15 μm. Abbreviations: Cp, cuticular projection; LaBa I–II, labial sensilla basiconica I–II; and LaTr I–II, labial sensilla trichodea I–II.

Labium of the sampled pentatomids is tubular, straight, and segmented by four, slender in plant feeders while stout in predators (Figure 1). A pair of the sensilla complex was laterally arranged on the labial apex, consisting of sensilla trichodea and sensilla basiconica (Figure 5 and Figure 6; Table 1) [29,48]. The length and basal diameter of the labial sensilla detected in the examined six species are provided in Tables S4–S9.

Labial sensilla trichodea (LaTr) are long, slender, and sharp tipped. Two subtypes of LaTr can be distinguished by size and distribution. Labial sensilla trichodea I (LaTr I) are long and marginally situated on the labial apex, while LaTr II are short and located in the labial sensilla complex (Figure 5 and Figure 6; Tables S5–S8).

Labial sensilla basiconica (LaBa) are peg-shaped. Three subtypes of Ba can be distinguished. Labial sensilla basiconica I (LaBa I), II (LaBa II), and III (LaBa III) have smooth walls, with LaBa I possessing rounded tips and common bases (Figure 5C,D and Figure 6). Labial sensilla basiconica II are morphologically similar to LaBa I, with the bases surrounded by inflexible sockets (Figure 5 and Figure 6). Labial sensilla basiconica III are only detected in *Eo*. *furcellata* and some other Asopinae species [4,30]; they have gradually tapered tips and swollen bases (Figure 5A,B) and possibly play an important role in prey detection and orientation.

Labial sensilla of heteropterans are crucial in responding to olfactory and gustatory signals, and excision of the labial apex strongly decreases the ability of prey orientation and capture efficiency [49]. In the present study, we found that the type and arrangement of labial sensilla seem to be differentiated between phytophagous and predatory species. Sensilla basiconica are the predominant type on the labial sensilla complex of plant feeding species, with sensilla trichodea singly scattered (Figure 5C,D and Figure 6) [4,12]. Interestingly, similar sensilla type and arrangement have been reported in many other plant feeders in Pentatomomorpha (e.g., chinch bugs, plant bugs, and lace bugs) [50,51,52], which implies the importance of labial sensilla on interacting with host plants. Species *Erthesina fullo* (Thunberg, 1783) [53] is an interesting exception, which is a phytophagous species with numerous sensilla trichodea clustered on the labial sensilla complex [48]. In predaceous stink bugs, sensilla trichodea and basiconica are the two primary types on the labial sensilla complex (Figure 5A,B) [30]. They are highly morphologically similar with the sensilla found in phytophagous stink bugs but are much larger in size than the same type of labial sensilla detected in typical predatory true bugs (e.g., assassin bugs and water striders) [7,54]. This morphological resemblance between predaceous and plant feeding stink bugs indicates the phylogenetic constraints on shaping labial sensilla in true bugs.

Moreover, the shape of labial cuticular projections varies greatly across pentatomid species with different feeding habits. They are short and slightly branched in plant feeders (Figure 5C,D and Figure 6A–D) but long and multi-branched in predators (Figure 5A,B) [4,29,30,49]. Although various function speculations of cuticular projections have been proposed (e.g., hygroreception, mechanoreception, and cleaning) [4,29,49], their actual utility remains to be tested experimentally. In *C. divaricata* and *S*. *lurida*, the labial apex of all of the examined samples was covered with contaminants and difficult to be cleaned to show the shape of labial sensilla and cuticular projections; therefore, the labial apex of these two species is not illustrated in the present study.

### 3.3. Modified Ultrastructure of Mandibles and Maxillae between Species with Different Feeding Habits

**Figure 7:**
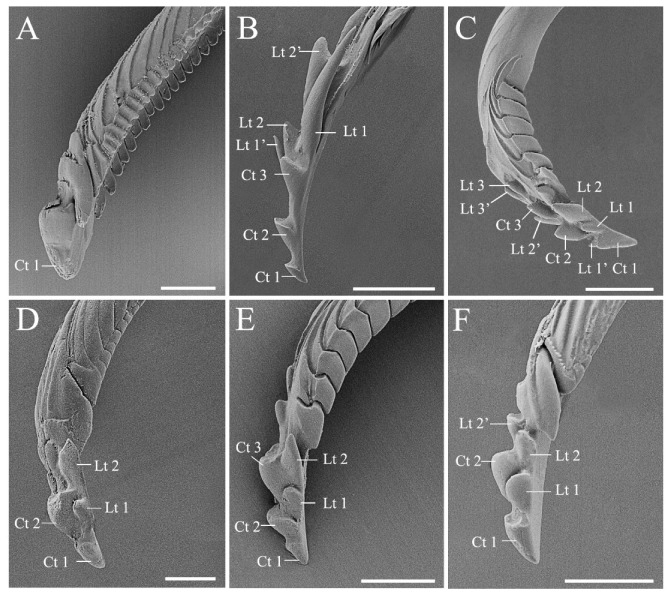
Mandibular stylet of the six pentatomid species. (**A**) *Cressona divaricata*, (**B**) *Eocanthecona furcellata*, (**C**) *Eurydema dominulus*, (**D**) *Halyomorpha halys*, (**E**) *Plautia crossota*, and (**F**) *Scotinophara lurida*. Scale bars: (**A**–**E**) 25 μm; (**F**) 15 μm. Abbreviations: Ct, central teeth; Lt, lateral teeth.

**Figure 8:**
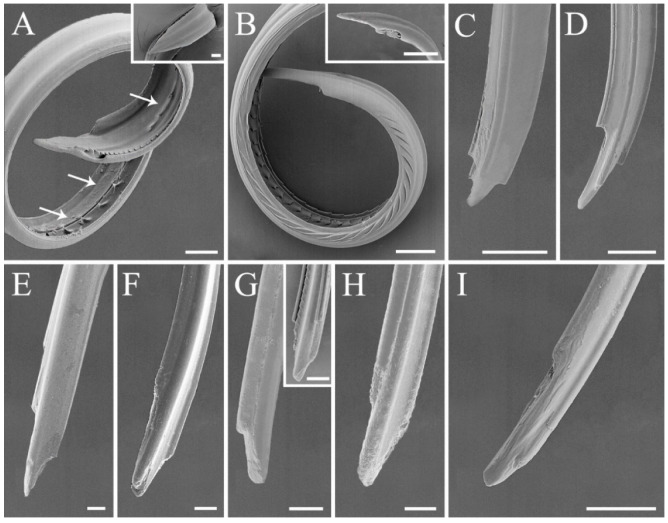
Maxillary stylets of the six pentatomid species. (**A**,**B**) *Eocanthecona furcellata*, with the external surface shown in the boxes and the arrows indicating the barbs; (**C**,**D**) *Eurydema dominulus*; (**E**,**F**) *Halyomorpha halys*; (**G**,**H**) *Plautia crossota*, with the internal surface shown in (**G**); (**I**) *Scotinophara lurida*. Scale bars: (**A**–**D**) 25 μm, (**A**,**B**) 20 μm, (**E**,**F**) 20 μm, (**G**–**I**) 15 μm, and (**G**) 25 μm.

Most of the stink bugs are phytophagous insects feeding mainly on seeds and immature fruits [55] in spite of twig or trunk feeding being reported in several species [56,57]. The subfamily Asopinae is an interesting exception, with all of the members being predators [17]. Feeding mechanism of pentatomids is composed of two main approaches, namely “salivary sheath” and “lacerate-and-flush” feeding, which is utilized by plant feeding and predaceous species, respectively. A sheath is formed by solidified gel saliva, surrounded the stylets to facilitate tissue penetration of the plant feeders. The predators use stylets to lacerate prey tissue to assist feeding [4,5,58,59]. Mandibles and maxillae (Figure 7 and Figure 8) are the principal parts of the feeding structures in Heteroptera [1,5]. In this study, we found significant morphological variations in mandibular teeth between species with distinct diet types. The stylet bundle of pentatomid species is composed of a pair of mandibles and maxillae (Figure 7 and Figure 8). They are long, thin and slender, bearing a strong inward curvature in the distal part in *Eo*. *furcellata* (Figure 7B and Figure 8A,B) and some other predatory stink bugs [9,30]. Mandibles laterally surround the maxillae, forming tubular concentric stylets completely enclosed by labium posterior-ventrally. The mandibular stylet was distally ornamented with scale-like patterns, and several central and lateral teeth. The central teeth are flattened, rounded, and broad, singly arrayed in all 17 species (Table 1) [4,9,30,48]. The lateral teeth are distinct in shape and arranged between phytophagous and predatory species. They tend to be short, blunted, approximately triangular, and symmetrically aligned in pairs in plant feeders (Figure 7A,C–F) [9,48], while in predatory species, they are sharp, elongated, hook-like, and irregularly arranged in predatory species (Figure 7B; Table 1) [4,30]. Additionally, the number of mandibular teeth varies among plant feeding species with different diet breaths. At least two central teeth and two pairs of lateral teeth are found in polyphagous herbivores (Figure 7C–F) [4,9,48], whereas single central tooth and no lateral tooth is found in the oligophagous *C*. *divaricata* (Figure 7A; Table S3). Future tests including more species with narrowed diet breath will help to better illustrate the association between tooth number and diet breath.

**Figure 9:**
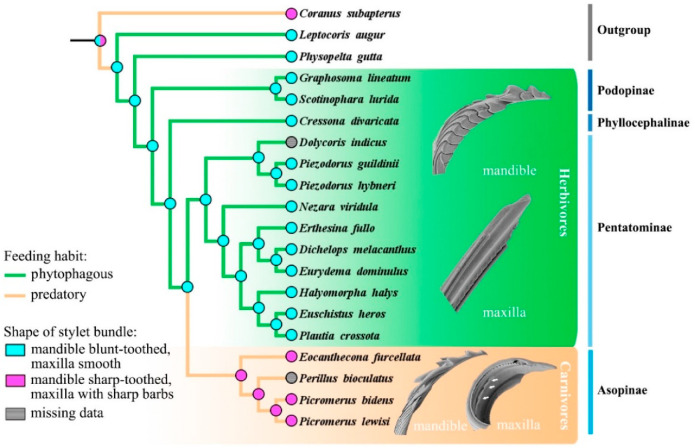
Ancestral state reconstruction of the shapes of mandibles and maxillae in Pentatomidae based on parsimony. The cladogram used for reconstruction was the single tree obtained based on the *cox1* and *rrnL* genes analyzed using the maximum likelihood method.

Maxillary stylets of the examined species are left–right asymmetrical, with one stylet narrower than the other on the distal end (Figure 8). This is consistent with previous descriptions on other stink bugs [4]. Maxillary stylets are interlocked, forming a food canal and a salivary canal, a typical shared characteristic of heteropterans [2,4]. A series of short and sharp tipped barbs are exclusively present on the food canal of predatory *Eo*. *furcellata* (Figure 8A and Figure 9), which is consistent with the observations from several other Asopinae species (Table 1) [4,30]. As a result, we suggest that ultrastructures from mandibular and maxillary stylets are the key characteristics associated with shift in feeding habits in stink bugs.

### 3.4. Ancestral States and Morphological Adaptations of Stink Bug Stylets

To trace the ancestral shape and state transformation of mandibular and maxillary characteristics in stink bugs, ancestral state reconstructions were conducted based on the single fully resolved topology resulting from the phylogenetic analysis (Figure 9). Two dominant types of stylet bundle were classified, with the herbivore type characterized as mandible blunt-toothed and maxilla smooth, possessed by the ancestors in Pentatomidae. In Asopinae, the shape of stylet bundle was modified into the carnivore type, characterized as mandibles with sharp or even hook-like teeth and maxillae with sharp barbs, which might be an adaptation to their carnivorous feeding habit evolved independently (Figure 9 and Figure S1). The mandibles of true bugs are often apically ornamented with various shaped teeth to facilitate penetration and anchoring of food [4,60]. It has been observed that the predatory stink bugs are capable of holding intensively struggling preys during predation [61] (e.g., personal observations of *Eo*. *furcellata* predating on fourth instar larvae of Asian corn borer); therefore, the sharp and hooked mandibular teeth present in the asopines might assist in the immobilization of living preys. Maxillary barbs were also documented in many other predaceous families and infraorders in Heteroptera (e.g., Reduviidae in Cimicomorpha; Hydrometridae in Gerromophora; and Belostomatidae, Nepidae, and Notonectidae in Nepomorpha) [4,30]. It is hypothesized that these barbs may serve to filter and triturate large-sized substrates for further digestion and absorption [4,5]. Considering that the ancestors of predatory stink bugs originated from plant feeding species in a largely phytophagous infraorder Pentatomomorpha [13,14,43], the presence of sharp mandibular teeth and maxillary barbs is likely to be a key innovation associated with transition from herbivorous to carnivorous feeding habit in Pentatomidae.

## 4. Conclusions

Overall, our study demonstrates that antennal and mouthpart ultrastructures vary remarkably across stink bug species with distinct feeding habits and, thus, might be key morphological characteristics driving feeding habit evolution in these insects. The types and arrangements of antennal and labial sensilla are different between predators and plant feeders, implying their crucial function in food orientation and selection. Particularly, we found that the mandibles are fringed with long and hook-like teeth and that the maxillae are ornamented with sharp barbs exclusively in predators. These ultrastructural modifications indicate their tight association with the shift in feeding habit from phytophagy to predation. Comparative morphological analysis involving a larger taxa sampling, together with the exploration of adaptive significance of these structural variations would shed light on how morphological evolution drives the adaptive diversification of feeding habits in these ecologically and economically important insect group.

## Figures and Tables

**Table 1:** Morphological similarity and disparity of antennae and labia between phytophagous and predatory stink bugs.

Morphological Characters	Similarity	Disparity
Antennal morphology	Five segments, including a proximal scape, a subdivided pedicel forming two segments, a basiflagellomere, and a distiflagellomere	N.A.
Antennal sensilla type and arrangement	Mainly four types, antennal sensilla chaetica, sensilla trichodea, sensilla basiconica, and sensilla coeloconica	A higher density of sensilla basiconica is observed on distiflagellomere in predatory *Eocanthecona furcellata* than in phytophagous species
Labial morphology	Tubular; straight; and segmented into four, namely labial segment I to IV from base to distal end	Slender in phytophagous species while stout in predators
Labial sensilla complex and arrangement	Mainly two types, labial sensilla trichodea and sensilla basiconica	Labial sensillum basiconica is the main type in phytophagous species (except for *Erthesina fullo*), while both sensillum trichodea and basiconica are the two main types in predators.
Labial cuticular projections	Present in phytophagous and predatory species	Short and slightly branched in phytophagous species, while long and multi-branched in predators
Mandibular stylet shape	With scale-like patterns, several central teeth, and lateral teeth; and the central teeth are flattened, rounded, and broad in phytophagous and predatory species	The lateral teeth are short, blunted, and symmetrically aligned in phytophagous species, while predators possess elongated and hook-like lateral teeth irregularly arranged on the mandible
Maxillary stylet shape	Left–right asymmetrical, with one stylet narrower than the other on the distal end	With short and sharp tipped barbs exclusively on the maxillae of predatory species

## Data Availability

The data generated in this study are provided here or in the Supplementary Materials, and they are also available upon request from the corresponding author.

## Supplementary Materials

The following are available online at https://www.mdpi.com/article/10.3390/biology10111161/s1, Figure S1, Ancestral state reconstruction of the shape of mandible (left) and maxilla (right) in Pentatomidae based on parsimony; Table S1, Genes accession numbers of *cox1* and *rrnL* for the species sampled in the present study; Table S2, Taxonomic and collected information on the stink bug species sampled in the present study; Table S3, Host plant information on the stink bug species sampled in the present study; Tables S4–S9, Morphometric data of labial and antennal sensilla of the six stink bug species examined in this study; Table S10, Average density of basiconica sensilla on antennal distiflagellomere of the stink bug species examined in the present study; Table S11, Morphological characteristics used for ancestral state reconstruction.
