# Supplementary material for: Ultrastructural Variations of Antennae and Labia Are Associated with Feeding Habit Shifts in Stink Bugs (Heteroptera: Pentatomidae)
Source: Biology (Basel). 2021 Nov 10;10(11):1161. doi: 10.3390/biology10111161 (PMC8615146; doi:10.3390/biology10111161)
Supplement: Supplementary file 1 [file biology-10-01161-s001.zip › biology-1435974-supplementary.pdf]

Supplementary Materials

Supplementary Figure S1

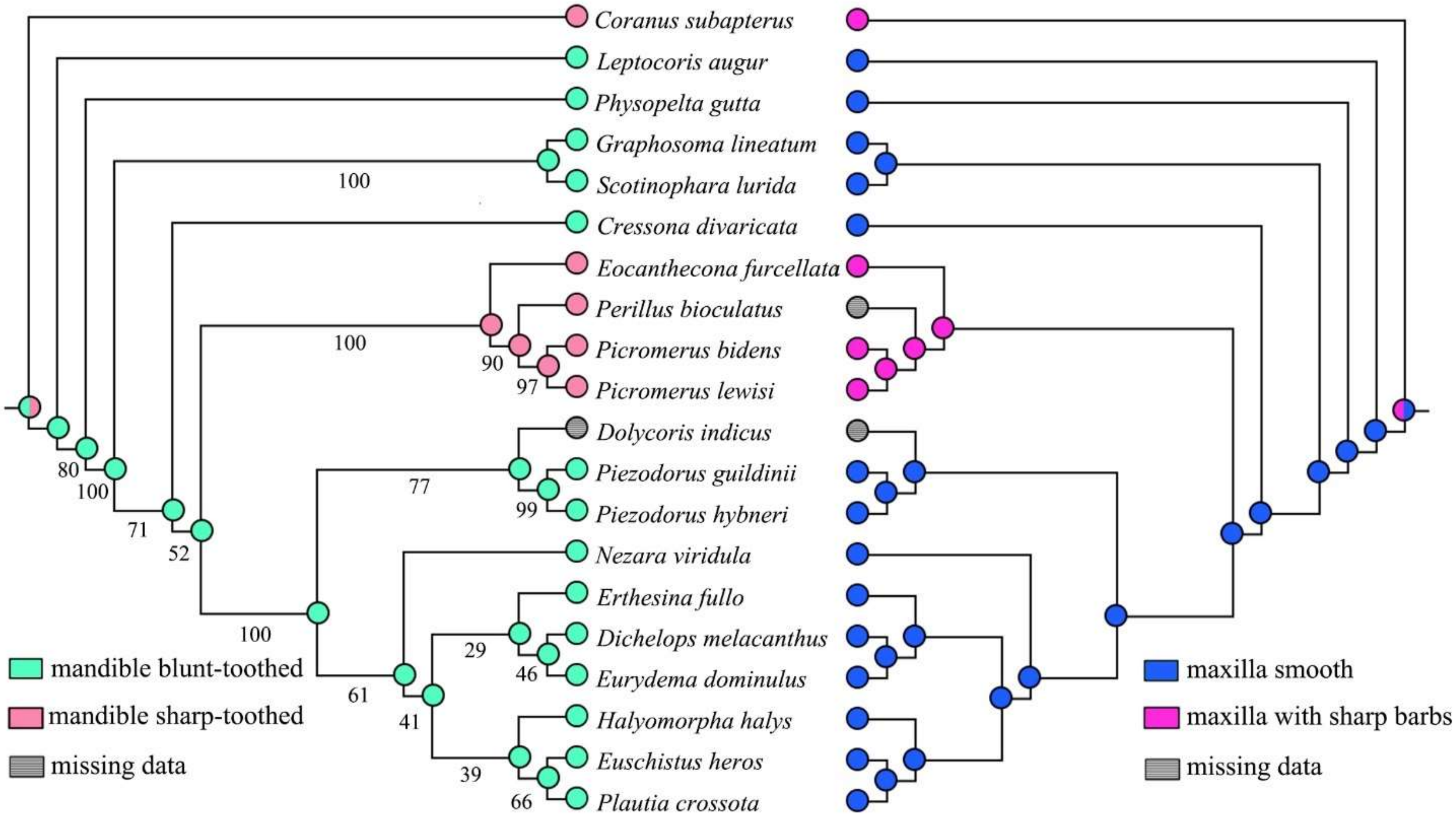

Figure S1. Ancestral state reconstruction of the shape of mandibles (left) and maxillae (right) in Pentatomidae based on parsimony. The cladogram used for reconstruction was the single tree obtained based on *cox1* and *rrnL* genes. The numbers under the branches are bootstrap values.

Table S1–S11

Table S1. Information of taxonomy, feeding habits, morphological references and genes *cox1* and *rrnL* accession numbers of all the species sampled in present study.

Family	Subfamily	Species	Feeding habit	Morphological reference	GenBank accession number	
					cox1	*rrnL*
Largidae Amyot & Serville	-	*Physopelta gutta* (Burmeister, 1834)[1]	Phytophagous	[2]	EU427343.1	EU427343.1
Reduviidae Latreille	Harpactorinae Amyot & Serville	*Coranus subapterus* (De Geer, 1773)[3]	Predatory	[4]	KM022149.1	-
Rhopalidae Amyot & Serville	-	*Leptocoris augur* (Fabricius, 1781)[5]	Phytophagous	[6]	KX503052.1	KX523378.1
					MK363262.1	
					KP142936.1	
Pentatomidae Leach	Phyllocephalinae Amyot & Serville	*Cressona divaricata* Zheng & Zou, 1982*[7]	Phytophagous	present study	MZ673416	MZ676042
	Asopinae Amyot & Serville	*Eocanthecona furcellata* (Wolff, 1811)[8]	Predatory	present study	MK393432.1	-
		Perillus bioculatus (Fabricius, 1775)[9]	Predatory	[4,10,11]	MG398669.1	-
		Picromerus bidens (Linnaeus, 1758)[12]	Predatory	[13]	KJ541616.1	MT265462.1
		Picromerus lewisi Scott, 1874[14]	Predatory	[13]	KY710772.1	KC155950.1
	Pentatominae Leach	*Dichelops melacanthus* Dallas, 1851[15]	Phytophagous	[16]	JQ218483.1<br>JQ218506.1<br>MN257052.1<br>JQ218458.1	KC537022.1
		Dolycoris indicus Stål, 1876[17]	Phytophagous	[10,11]	KX467344.1	-
		Erthesina fullo (Thunberg, 1783)[18]	Phytophagous	[19]	MK374364.1	MK374364.1
		Eurydema dominulus (Scopoli, 1763)[20]	Phytophagous	present study	MG584833.1	MG584833.1
		Euschistus heros (Fabricius, 1798)[21]	Phytophagous	[16,22]	MN257053.1<br>KU892543.1	KU853769.1

	Halyomorpha halys (Stål, 1855)[23]	Phytophagous	present study	NC_013272.1	NC_013272.1
	Nezara viridula (Linnaeus, 1758)[12]	Phytophagous	[16,24,25]	NC_011755.1	NC_011755.1
	Piezodorus guildinii (Westwood, 1837)[26]	Phytophagous	[16,22]	HQ985132.1<br>JX425420.1<br>JQ218490.1<br>JQ218510.1	JX425407.1
	Piezodorus hybneri (Gmelin, 1790)[27]	Phytophagous	[10,16]	KX467346.1	-
	Plautia crossota (Dallas, 1851)[15]	Phytophagous	present study	MK757497.1	MK757497.1
Podopinae Amyot & Serville	*Graphosoma lineatum* (Linnaeus, 1758) [12]	Phytophagous	[4]	KM021441.1	-
				KX960066.1	
	Scotinophara lurida (Burmeister, 1834)[1]	Phytophagous	present study	MF497733.1	MF497733.1

* Fragments of *cox1* and *rrnL* sequenced in present study.

Table S2. Taxonomic and collecting information of the stink bug species examined in present study.

Subfamily	Species	Locality	Date
Phyllocephalinae Amyot & Serville	*Cressona divaricata* Zheng & Zou, 1982	Qimaba, Honghe, Yunan, China	2012-V-20
Asopinae Amyot & Serville	*Eocanthecona furcellata* (Wolff, 1811)	Wenshan, Yunan, China	2020-VII-27
Pentatominae Leach	*Eurydema dominulus* (Scopoli, 1763)	Qifang Town, Xiangyang, Hubei, China	2018-VI-14
	Halyomorpha halys (Stål, 1855)	Haidian, Beijing, China	2018-X-23
	Plautia crossota (Dallas, 1851)	Tangfu County, HongKong, China	2019-V-12
Podopinae Amyot & Serville	*Scotinophara lurida* (Burmeister, 1834)	Fengtongzhai National Nature Reserve, Yaan, Sichuan, China	2018-VIII-29

Table S3. Host plant information of the stink bug species sampled in present study.

Subfamily	Species	Host plant family*
Phyllocephalinae Amyot & Serville	*Cressona divaricata* Zheng & Zou, 1982	Poaceae
Pentatominae Leach	*Dichelops melacanthus* Dallas, 1851	Fabaceae
		Oleaceae
		Poaceae
	Dolycoris indicus Stål, 1876	Apiaceae
		Asteraceae
		Brassicaceae
		Cannabaceae
		Chenopodiaceae
		Cuscutaceae
		Fabaceae
		Liliaceae
		Linaceae
		Malvaceae
		Plantaginaceae
		Poaceae
		Polygonaceae
		Santalaceae
		Solanaceae
	Erthesina fullo (Thunberg, 1783)	Anacardiaceae
		Apocynaceae
		Araliaceae
		Bignoniaceae
		Burseraceae
		Cannabinaceae
		Casuarinaceae
		Cupressaceae
		Ebenaceae
		Euphorbiaceae
		Fabaceae
		Hamamelidaceae
		Juglandaceae
		Lauraceae
		Magnoliaceae
		Malvaceae
		Meliaceae
		Mimosaceae
		Moraceae
		Myrtaceae
		Oxalidaceae
		Pinaceae
		Platanaceae
		Poaceae
		Proteaceae
		Punicaceae
		Rhamnaceae
		Rosaceae
		Rubiaceae
		Salicaceae
		Santalaceae
		Scrophulariaceae
		Simaroubaceae
		Ulmaceae
		Verbenaceae

Eurydema dominulus (Scopoli, 1763)	Apiaceae
	Balsaminaceae
	Brassicaceae
	Lamiaceae
	Scrophulariaceae
Euschistus heros (Fabricius, 1798)	Brassicaceae
	Fabaceae
	Oleaceae
	Solanaceae
Halyomorpha halys (Stål, 1855)	Amaranthaceae
	Basellaceae
	Ebanaceae
	Fabaceae
	Malvaceae
	Moraceae
	Oleaceae
	Rosaceae
	Rutaceae
	Solanaceae
	Vitaceae
Nezara viridula (Linnaeus, 1758)	Amaranthaceae
	Anacardiaceae
	Apiaceae
	Aquifoliaceae
	Asteraceae
	Brassicaceae
	Cannabaceae
	Capparaceae
	Chenopodiaceae
	Cupressaceae
	Euphorbiaceae
	Fagaceae
	Lamiaceae
	Liliaceae
	Linaceae
	Malvaceae
	Meliaceae
	Mimosaceae
	Oleaceae
	Pedaliaceae
	Plantaginaceae
	Poaceae
	Polygonaceae
	Rosaceae
	Rutaceae
	Scrophulariaceae
	Solanaceae
Piezodorus guildinii (Westwood, 1837)	Apiaceae
	Aquifoliaceae
	Brassicaceae
	Cucurbitaceae
	Fabaceae
	Linaceae
	Malpighiaceae
	Oleaceae
	Phytolaccaceae
	Rosaceae

		Solanaceae
	Piezodorus hybneri (Gmelin, 1790)	Anacardiaceae
		Fabaceae
		Poaceae
		Verbenaceae
	Plautia crossota (Dallas, 1851)	Anacardiaceae
		Asteraceae
		Basellaceae
		Brassicaceae
		Cannaceae
		Caprifoliaceae
		Convolvulaceae
		Fabaceae
		Moraceae
		Poaceae
		Rhamnaceae
		Rubiaceae
		Santalaceae
		Scrophulariaceae
		Solanaceae
		Tiliaceae
		Verbenaceae
Podopinae Amyot & Serville	*Graphosoma lineatum* (Linnaeus, 1758)	Apiaceae
		Asteraceae
	Scotinophara lurida (Burmeister, 1834)	Cyperaceae
		Marsileaceae
		Poaceae
		Pontederiaceae

Information of host plant family was summarized from the online database Pentatomoidea Home Page, with the related literatures available on https://www.ndsu.edu/pubweb/~rider/Pentatomoidea/.

Table S4. Morphometric data of labial and antennal sensilla in *Cressona divaricata* Zheng & Zou, 1982.

Sensilla type	Length (μm)	Basal Diameter (μm)	Distribution
AnCh I	71.62 ± 11.88	6.37 ± 1.84	Sc, BPd, DPd, Bf, Df.
AnTr I	58.67 ± 6.70	4.25 ± 0.66	Bf, Df.
AnTr II	44.80 ± 5.23	2.73 ± 0.45	Bf, Df.
AnBa I	15.28 ± 0.88	2.40 ± 0.26	Bf, Df.

Data are mean ± SD values obtained from scanning electron microscopy based on about 20 antennal sensilla of each type selected from antennal scape to distiflagellomere of males and females. AnBa I, antennal sensilla basiconica I; AnCh I, antennal sensilla chaetica I; AnTr I–II, antennal sensilla trichodea I–II; Bf, basiflagellomere; BPd, basal pedicel; Df, distiflagellomere; DPd, distal pedicel; Sc, scape.

Table S5. Morphometric data of labial and antennal sensilla in *Eocanthecona furcellata* (Wolff, 1811).

Sensilla type	Length (μm)	Basal Diameter (μm)	Distribution
AnCh I	44.62 ± 2.50	4.16 ± 0.67	Sc, BPd, DPd, Bf, Df.
AnCh II	43.18 ± 8.01	3.38 ± 0.69	Sc, BPd.
LaTr I	44.59 ± 8.21	2.14 ± 0.63	La.
LaTr II	21.94 ± 2.79	3.16 ± 0.99	La.
AnTr I	47.77 ± 2.93	3.43 ± 0.87	DPd, Bf, Df.

AnTr II	37.67 ± 1.08	1.41 ± 0.15	DPd, Bf, Df.
LaBa I	19.86 ± 1.22	3.14 ± 0.63	La.
LaBa II	7.07 ± 2.02	2.19 ± 0.24	La.
LaBa III	22.15 ± 1.68	8.36 ± 1.91	DPd, Bf, Df.
AnBa I	13.15 ± 2.75	1.93 ± 0.10	DPd, Bf, Df.
AnCo I	2.37 ± 0.08	1.63 ± 0.05	Df.
AnCo II	-	0.35 ± 0.06	Df.

Data are mean ± SD values obtained from scanning electron microscopy based on about 20 antennal sensilla of each type selected from antennal scape to distiflagellomere of males and females. Antennal sensilla coeloconica I and II are tiny in size, few in number and sparsely scattered on distiflagellomere; therefore, their measurements were got from all the observed ones (around 3 to 5). AnBa I, antennal sensilla basiconica I; AnCh I–II, antennal sensilla chaetica I–II; AnCo I–II, antennal sensilla coeloconica I–II; AnTr I–II, antennal sensilla trichodea I–II; LaBa I–III, labial sensilla basiconica I–III; Bf, basiflagellomere; BPd, basal pedicel; Df, distiflagellomere; DPd, distal pedicel; LaBa I–III, labial sensilla basiconica I–III; LaTr I–II, labial sensilla trichodea I–II; Sc, scape.

Table S6. Morphometric data of labial and antennal sensilla in *Eurydema dominulus* (Scopoli, 1763).

Sensilla type	Length (μm)	Basal Diameter (μm)	Distribution
AnCh I	46.80 ± 5.50	4.22 ± 0.24	Sc, BPd, DPd, Bf, Df.
LaTr I	44.01 ± 4.52	3.54 ± 0.12	La.
LaTr II	23.01 ± 1.65	3.42 ± 0.30	La.
AnTr I	34.40 ± 2.71	3.23 ± 0.43	Bf, Df.
AnTr II	32.36 ± 2.83	1.51 ± 0.29	Bf, Df.
LaBa I	11.97 ± 1.61	3.36 ± 0.14	La.
LaBa II	6.93 ± 0.82	2.52 ± 0.30	La.
AnBa I	10.87 ± 0.92	2.46 ± 0.30	Bf, Df.

Data are mean ± SD values obtained from scanning electron microscopy based on about 20 antennal sensilla of each type selected from antennal scape to distiflagellomere of males and females. AnBa I, antennal sensilla basiconica I; AnCh I, antennal sensilla chaetica I; AnTr I–II, antennal sensilla trichodea I–II; Bf, basiflagellomere; BPd, basal pedicel; Df, distiflagellomere; DPd, distal pedicel; LaBa I–II, labial sensilla basiconica I–II; LaTr I–II, labial sensilla trichodea I–II; Sc, scape.

Table S7. Morphometric data of labial and antennal sensilla in *Halyomorpha halys* (Stål, 1855).

Sensilla type	Length (μm)	Basal Diameter (μm)	Distribution
AnCh I	52.12 ± 7.49	4.38 ± 0.62	Sc, BPd, DPd, Bf, Df.
LaTr I	38.60 ± 9.17	2.85 ± 0.26	La.
LaTr II	24.11 ± 9.36	2.49 ± 0.07	La.
AnTr I	49.03 ± 5.40	3.51 ± 0.45	BPd, DPd, Bf, Df.
AnTr II	38.68 ± 2.28	1.74 ± 0.41	BPd, DPd, Bf, Df.
LaBa I	12.95 ± 2.80	3.59 ± 0.22	La.
LaBa II	6.44 ± 1.70	2.95 ± 0.33	La.
AnBa I	19.50 ± 1.09	2.30 ± 0.20	DPd, Bf, Df.
AnBa II	11.74 ± 1.08	2.48 ± 0.10	DPd, Bf, Df.

AnCo I	3.71 ± 0.03	3.35 ± 0.20	Df.

Data are mean ± SD values obtained from scanning electron microscopy based on about 20 antennal sensilla of each type selected from antennal scape to distiflagellomere of males and females. Antennal sensilla coeloconica I are tiny in size, few in number and sparsely scattered on distiflagellomere; therefore, their measurements were got from all the observed ones (around 3 to 5). AnBa I–II, antennal sensilla basiconica I–II; AnCh I, antennal sensilla chaetica I; AnCo I, antennal sensilla coeloconica I; AnTr I–II, antennal sensilla trichodea I–II; Bf, basiflagellomere; BPd, basal pedicel; Df, distiflagellomere; DPd, distal pedicel; LaBa I–II, labial sensilla basiconica I–II; LaTr I–II, labial sensilla trichodea I–II; Sc, scape.

Table S8. Morphometric data of labial and antennal sensilla in *Plautia crossota* (Dallas, 1851).

Sensilla type	Length (μm)	Basal Diameter (μm)	Distribution
AnCh I	61.95 ± 6.17	4.16 ± 0.76	Sc, BPd, DPd, Bf, Df.
LaTr I	56.28 ± 5.49	2.76 ± 0.23	La.
LaTr II	20.47 ± 1.29	3.10 ± 0.60	La.
AnTr I	41.75 ± 6.08	3.11 ± 0.12	DPd, Bf, Df.
AnTr II	35.98 ± 3.09	1.66 ± 0.26	DPd, Bf, Df.
LaBa I	14.37 ± 0.75	3.89 ± 0.22	La.
LaBa II	10.96 ± 1.12	3.14 ± 0.23	La.
AnBa I	17.51 ± 1.21	1.48 ± 0.15	DPd, Bf, Df.
AnBa II	11.65 ± 0.83	1.93 ± 0.30	DPd, Bf, Df.
AnCo I	2.07 ± 0.08	1.43 ± 0.09	Df.
AnCo II	-	0.54 ± 0.02	Df.

Data are mean ± SD values obtained from scanning electron microscopy based on about 20 antennal sensilla of each type selected from antennal scape to distiflagellomere of males and females. Antennal sensilla coeloconica I and II are tiny in size, few in number and sparsely scattered on distiflagellomere; therefore, their measurements were got from all the observed ones (around 3 to 5). AnBa I–II, antennal sensilla basiconica I–II; AnCh I, antennal sensilla chaetica I; AnCo I–II, antennal sensilla coeloconica I–II; AnTr I–II, antennal sensilla trichodea I–II; Bf, basiflagellomere; BPd, basal pedicel; Df, distiflagellomere; DPd, distal pedicel; LaBa I–II, labial sensilla basiconica I–II; LaTr I–II, labial sensilla trichodea I–II; Sc, scape.

Table S9. Morphometric data of labial and antennal sensilla in *Scotinophara lurida* (Burmeister, 1834).

Sensilla type	Length (μm)	Basal Diameter (μm)	Distribution
AnCh I	63.48 ± 4.63	3.17 ± 0.42	Sc, BPd, DPd, Bf, Df.
AnTr I	67.85 ± 9.17	3.69 ± 0.63	Bf, Df.
AnTr II	43.81 ± 3.41	2.09 ± 0.54	Bf, Df.
AnBa I	17.59 ± 2.43	2.30 ± 0.40	Bf, Df.
AnCo II	-	0.90 ± 0.09	Df.

Data are mean ± SD values obtained from scanning electron microscopy based on about 20 antennal sensilla of each type selected from antennal scape to distiflagellomere of males and females. Antennal sensilla coeloconica II are tiny in size, few in number and sparsely scattered on distiflagellomere; therefore, their measurements were got from all the observed ones (around 3 to 5). AnBa I, antennal sensilla basiconica I; AnCh I, antennal sensilla chaetica I; AnCo II, antennal sensilla coeloconica II; AnTr I–II, antennal sensilla trichodea I–II; Bf, basiflagellomere; BPd, basal pedicel; Df, distiflagellomere; DPd, distal pedicel; Sc, scape.

Table S10. Average density of basiconica sensilla on antennal distiflagellomere of the stink bug species examined in present study.

Species	Density (/10^4 μm^2)
Cressona divaricata Zheng & Zou, 1982	1.25 ± 0.32
Eocanthecona furcellata (Wolff, 1811)	22.63 ± 7.48
Eurydema dominulus (Scopoli, 1763)	3.10 ± 1.67
Halyomorpha halys (Stål, 1855)	5.10 ± 0.76
Plautia crossota (Dallas, 1851)	7.74 ± 1.88
Scotinophara lurida (Burmeister, 1834)	5.59 ± 1.66

Data are mean ± SD values obtained from scanning electron microscopy. The densities were calculated by averaging the number of basiconica sensilla in two randomly selected 1000 μm^3 quadrats on the electron microscopic images from males and females.

Table S11. Morphological characters used for ancestral state reconstruction.

Family	Subfamily	Species	Mandible (0: sharp-toothed; 1: blunt toothed)	Maxilla (0: smooth; 1: with sharp barbs)	Reference
Largidae Amyot & Serville	-	*Physopelta gutta* (Burmeister, 1834)	0	0	[2]
Reduviidae Latreille	Harpactorinae Amyot & Serville	*Coranus subapterus* (De Geer, 1773)	1	1	[4]
Rhopalidae Amyot & Serville	-	*Leptocoris augur* (Fabricius, 1781)	0	0	[6]
Pentatomidae Leach	Phyllocephalinae Amyot & Serville	*Cressona divaricata* Zheng & Zou, 1982	0	0	present study
	Asopinae Amyot & Serville	*Eocanthecona furcellata* (Wolff, 1811)	1	1	present study
		Perillus bioculatus (Fabricius, 1775)	1	?	[4]
		Picromerus bidens (Linnaeus, 1758)	1	1	[13]
		Picromerus lewisi Scott, 1874	1	1	[13]
	Pentatominae Leach	*Dichelops melacanthus* Dallas, 1851	0	0	[16]
		Dolycoris indicus Stål, 1876	?	?	N.A. (with antennal characters available for this species)

Erthesina fullo (Thunberg, 1783)	0	0	[19]
Eurydema dominulus (Scopoli, 1763)	0	0	present study
Euschistus heros (Fabricius, 1798)	0	0	[16]
Halyomorpha halys (Stål, 1855)	0	0	present study
Nezara viridula (Linnaeus, 1758)	0	0	[16]
Piezodorus guildinii (Westwood, 1837)	0	0	[16]
Piezodorus hybneri (Gmelin, 1790)	0	0	[16]
Plautia crossota (Dallas, 1851)	0	0	present study
Graphosoma lineatum (Linnaeus, 1758)	0	0	[4]
Scotinophara lurida (Burmeister, 1834)	0	0	present study

Reference

1. Burmeister, H. Rhyngota seu Hemiptera. In: Meyen, F.J.F Beiträge zur Zoologie, gesammelt auf einer Reise um die Erde, und W. Erichson's und H. Burmeister's Beschreibungen und Abbildungen der von Herrn Meyen auf dieser Reise gesammelten Insekten. *Nov. Acta Acad. Caesareae Leopoldino-Carolinae Naturae Curiosorum* **1834**, *16*, 285–308.
2. Wang, Y.; Brożek, J.; Dai, W. Morphological disparity of the mouthparts in polyphagous species of Largidae (Heteroptera: Pentatomomorpha: Pyrrhocoroidea) reveals feeding specialization. *Insects* **2020**, *11*, 145, doi:10.3390/insects11030145.
3. De Geer, C. *Mémoires pour servir à l'histoire des insectes*; Pierre Hesselberg: Stockholm, 1773; Vol. 3, pp. 1–696.
4. Cobben, R.H. Evolutionary trends in Heteroptera. Part 2. Mouth part structures and feeding strategies. *Meded. Landbouwhogesch. Wagening* **1978**, *78*, 5–407.
5. Fabricius, J.C. *Species insectorum exhibentes eorum differentias specificas, synonyma auctorum, loca natalia, metamorphosin adjectis observationibus, descriptionibus*; Bohnii: Hamburgi et Kilonii, 1781; p. 366.
6. Badwaik, V.; Barsagade, D. Distribution of sensilla and interlocking of mouthparts in red eye bug, *Leptocoris augur* (Hemiptera: Heteroptera: Rhopalidae). *Int. J. Res. Biosci. Agric. Technol.* **2014**, *2*, 193–207.
7. Zheng, L.Y.; Zou, H.G. Records of heteropterous insects on bamboo from Yunnan. *Zool. Res.* **1982**, *3*, 113–120.
8. Wolff, J.F. *Icones Cimicum descriptionibus illustratae*; Palm: Erlangen, 1811; Vol. 5, p. 182.
9. Fabricius, J.C. *Systema entomologiae sistens insectorum classes, ordines, genera, species; adjectis synonymis, locis, descriptionibus et observationibus*; Kortii: Flensburgi et Lipsiae, 1775; pp. 1–832.
10. Parveen, S.; Ahmad, A.; Broek, J.; Ramamurthy, V.V. Morphological diversity of the labial sensilla of phytophagous and predatory Pentatomidae (Hemiptera: Heteroptera), with reference to their possible functions. *Zootaxa* **2015**, *4039*, 359–372, doi:10.11646/zootaxa.4039.2.9.
11. Ahmad, A.; Parveen, S.; Brożek, J.; Dey, D. Antennal sensilla of phytophagous and predatory pentatomids

(Hemiptera: Pentatomidae): a comparative study of four genera. *Zool. Anz.* **2016**, *261*, 48–55, doi:10.1016/j.jcz.2016.03.007.

12. Linnaeus, C. *Systema naturae per regna tria naturae: secundum classes, ordines, genera, species, cum characteribus, differentiis, synonymis, locis. Editio decima, reformata*; Salvii: Stockholm, 1758; pp. 442–444.
13. Wang, Y.; Brożek, J.; Dai, W. Comparative morphology of the mouthparts in three predatory stink bugs (Heteroptera: Asopinae) reveals feeding specialization of stylets and sensilla. *Insects* **2020**, *11*, 1–23, doi:10.3390/insects11110762.
14. Scott, J. On a collection of Hemiptera Heteroptera from Japan. Descriptions of various new genera and species. *Ann. Mag. Nat. Hist.* **1874**, *14*, 289–304.
15. Dallas, W.S. *List of the specimens of hemipterous insects in the collection of the British Museum. Part I*; Trustees of the British Museum: London, 1851; pp. 1–368.
16. Depieri, R.A.; Panizzi, A.R. Rostrum length, mandible serration, and food and salivary canals areas of selected species of stink bugs (Heteroptera, Pentatomidae). *Rev. Bras. Entomol.* **2010**, *54*, 584–587, doi:10.1590/S0085-56262010000400008.
17. Stål, C. *Enumeratio hemipterorum : bidrag till en förteckning öfver alla hittills kända hemiptera, jemte systematiska meddelanden*; P. A. Norstedt: Stockholm, 1876; Vol. 14; p. 76.
18. Thunberg, C.P. *Dissertatio entomologica novas insectorum species, sistens, cujus partem secundum*; Edman: Upsaliae, 1783; p. 42.
19. Wang, Y.; Dai, W. How does the intricate mouthpart apparatus coordinate for feeding in the hemimetabolous insect pest *Erthesina fullo*? *Insects* **2020**, *11*, 1–24, doi:10.3390/insects11080503.
20. Scopoli, J.A. *Entomologia carniolica exhibens Insecta Carnioliae indigena et distributa in ordines, genera, species, varietatis. Methodo Linnaeana*; Trattner: Vindobonae, 1763; p. 124.
21. Fabricius, J.C. *Entomologia systematica emendata et aucta, secundum classes, ordines, genera, species, adjectis synonymis, locis, observationibus*; Proft et Storch: Hafniae, 1798; pp. 1–572.
22. Silva, C.C.A.; de Capdeville, G.; Moraes, M.C.B.; Falcão, R.; Solino, L.F.; Laumann, R.A.; Silva, J.P.; Borges, M. Morphology, distribution and abundance of antennal sensilla in three stink bug species (Hemiptera: Pentatomidae). *Micron* **2010**, *41*, 289–300, doi:10.1016/j.micron.2009.11.009.
23. Stål, C. *Öfversigt af Kongl. Vetenskaps-akademiens forhandlingar*; P. A. Norstedt & Söner: Stockholm, 1855; Vol. 12; p.182.
24. Rani, P.U.; Madhavendra, S.S. Morphology and distribution of antennal sense organs and diversity of mouthpart structures in *Odontopus nigricornis* (Stål) and *Nezara viridula* L. (Hemiptera). *Int. J. Insect Morphol. Embryol.* **1995**, *24*, 119–132, doi:10.1016/0020-7322(94)00020-Q.
25. Brézot, P.; Tauban, D.; Renou, M. Sense organs on the antennal flagellum of the green stink bug, *Nezara viridula* (L.) (Heteroptera: Pentatomidae) : sensillum types and numerical growth during the post-embryonic development. *Int. J. Insect Morphol. Embryol.* **1996**, *25*, 427–441, doi:10.1016/S0020-7322(96)00012-8.
26. Westwood, J.O. *A catalogue of Hemiptera in the collection of the Rev. F. W. Hope, M. A. with short Latin diagnoses of the new species*; J. C. Bridgewater: London, 1837; p. 7.
27. Gmelin, J.F. *Caroli a Linné Systema Naturae*; Beer: Lipsiae, 1790; p. 2151.
